# Therapeutic Potential of Selected Flavonoids as Natural Antidiabetic Agents

**DOI:** 10.3390/nu18182944

**Published:** 2026-09-08

**Authors:** Klaudia Stocerz, Arkadiusz Sokal, Patryk Mruczek, Monika Kadela-Tomanek, Paweł Ramos

**Affiliations:** 1 Doctoral School, Medical University of Silesia in Katowice, 15 Poniatowskiego Str., 40-055 Katowice, Poland; klaudia.stocerz@sum.edu.pl (K.S.); arkadiusz.sokal@sum.edu.pl (A.S.); 2 Department of Community Pharmacy, Faculty of Pharmaceutical Sciences in Sosnowiec, Medical University of Silesia in Katowice, 8b Jednosci Str., 41-200 Sosnowiec, Poland; pawelramos@sum.edu.pl; 3 Department of Organic Chemistry, Faculty of Pharmaceutical Sciences in Sosnowiec, Medical University of Silesia, 4 Jagiellonska Str., 41-200 Sosnowiec, Poland; s84812@365.sum.edu.pl

**Keywords:** type 2 diabetes mellitus, postprandial hyperglycemia, flavonoids, α-amylase, α-glucosidase

## Abstract

Type 2 diabetes mellitus (T2DM) remains a major clinical challenge, with postprandial hyperglycemia (PPG) playing a key role in the development of vascular complications. Current pharmacotherapies often do not sufficiently control PPG, highlighting the need for complementary strategies. One effective approach is the inhibition of carbohydrate digesting enzymes, α-amylase and α-glucosidase, which delays glucose absorption and reduces PPG excursions. Flavonoids, a diverse class of plant-derived polyphenols, exhibit multiple biological activities relevant to T2DM, including enzyme inhibition, antioxidant, and anti-inflammatory effects. This review summarizes the mechanisms by which flavonoids modulate glucose metabolism, with particular emphasis on their interactions with α-amylase and α-glucosidase. Structure–activity relationships are discussed, focusing on the influence of hydroxylation patterns, conjugation, and molecular planarity on inhibitory potency and selectivity. Selected flavonoids—chrysin, apigenin, luteolin, and quercetin—are comparatively analyzed in terms of enzyme inhibition profiles, binding mechanisms, and selectivity. Evidence from in vitro studies, enzyme kinetics, and molecular docking highlights their differential activity, particularly the preferential inhibition of α-glucosidase over α-amylase, which may reduce gastrointestinal side effects. Overall, flavonoids represent promising modulators of PPG. However, their clinical relevance is limited by bioavailability and variability of experimental data, warranting further investigation.

## 1. Introduction

### 1.1. Clinical Background and Role of Postprandial Hyperglycemia

In the presence of the significant therapeutic progress in recent years, type 2 diabetes mellitus (T2DM) remains a condition in which achieving optimal metabolic control poses a significant clinical challenge. Despite the availability of numerous drug classes and diverse therapeutic regimens, maintaining stable glycemia in many patients remains difficult in clinical practice [1]. One significant factor affecting treatment effectiveness is poor adherence to treatment recommendations, which is reflected in the high percentage of patients who are overweight or obese, exceeding 80%. Low adherence contributes to variability in treatment response and often necessitates intensification of therapy, including the introduction of insulin, which indicates difficulties in maintaining adequate glycemic control [2,3,4,5].

A significant, yet often inadequately controlled, component of the clinical picture of T2DM is PPG, which plays a key role in the development of vascular complications, regardless of fasting blood glucose levels or glycated hemoglobin (HbA1c) levels [6]. Repeated, rapid increases in glucose concentration after meals lead to increased oxidative stress through increased generation of reactive oxygen species (ROS), resulting in endothelial cell damage. This leads to impaired vascular function, activation of inflammatory processes, and increased expression of adhesion molecules, which promotes the initiation and progression of atherosclerotic lesions. Furthermore, glycemic variability intensifies non-enzymatic protein glycation processes and leads to the formation of advanced glycation end products (AGEs), which damage the structure of the vascular wall and contribute to the development of microvascular and macrovascular complications [7,8,9].

Particularly unfavorable consequences are observed in patients with obesity, who often present with chronic low-grade inflammation, severe insulin resistance, and impaired adipose tissue function. These factors further exacerbate endothelial damage and accelerate the progression of vascular changes. Consequently, the risk of developing complications is significantly increased and includes accelerated progression of atherosclerosis, coronary artery disease, stroke, and microvascular complications such as retinopathy, nephropathy, and diabetic neuropathy [10,11,12,13,14]. Therefore, PPG is considered a significant, independent risk factor for cardiovascular disease, particularly in patients with excess body weight [15,16,17].

In contrast to many commonly used therapeutic strategies, such as the use of basal insulins [18] or metformin, which largely focus on the regulation of fasting glucose, PPG control remains inadequate. This limits their impact on reducing the risk of vascular complications associated with PPG fluctuations. One approach to more targeted intervention is to modulate the activity of digestive enzymes involved in carbohydrate metabolism. A key role is played by α-glucosidase and α-amylase, responsible for the hydrolysis of complex carbohydrates into forms that can be absorbed in the small intestine [19].

Inhibiting the activity of these enzymes is an effective strategy for directly controlling PPG by slowing the rate of glucose absorption from the gastrointestinal tract, which leads to a flattening of the PPG profile and limits its harmful effects on the vascular system. This approach enables intervention at an early stage of glucose metabolism, which promotes greater stability of the therapeutic effect, also in conditions of limited patient adherence [20,21,22].

Acarbose remains the most commonly used substance modulating the activity of these enzymes in T2DM therapy, alongside compounds such as voglibose and miglitol. Acarbose, by inhibiting α-glucosidase and α-amylase, effectively reduces PPG and is a reference point in research on new digestive enzyme inhibitors [23,24,25]. However, its clinical use is limited by common gastrointestinal side effects such as flatulence, diarrhea and intestinal discomfort. These symptoms result from the increased amount of undigested carbohydrates available to the intestinal microbiota, which intensifies fermentation processes. As a consequence, treatment tolerance is low in some patients, which negatively affects compliance with therapeutic recommendations. Furthermore, the need to maintain specific dosing regimens to limit side effects increases the complexity of therapy and requires greater involvement from both the patient and healthcare team [26,27,28,29,30,31,32,33,34].

One solution to reduce the described problems is the use of combination therapy, including acarbose in combination with compounds of natural origin, to improve treatment tolerance. In this paper, particular attention is paid to flavonoids as substances with documented, multifaceted biological activity. Their ability to inhibit α-glucosidase and α-amylase is discussed in detail later in the manuscript; however, their broad spectrum of action in processes relevant to the pathophysiology of metabolic diseases should be emphasized here.

The pleiotropic properties of flavonoids, including anti-inflammatory activity, may contribute to reducing gastrointestinal symptoms by modulating local inflammation in the intestines. Furthermore, the ability of these compounds to simultaneously affect multiple metabolic pathways enhances their therapeutic potential [35,36,37,38]. In contrast to acarbose, which works by simultaneously inhibiting α-glucosidase and α-amylase, the more selective inhibition of α-glucosidase—with a relatively weaker effect on α-amylase—may be associated with less severe side effects such as diarrhea [38,39,40,41].

Considering their multifaceted mechanism of action and favorable safety profile, flavonoids constitute a promising group of natural α-glucosidase inhibitors, which warrants further analysis of their therapeutic potential in the following sections of this article.

### 1.2. Flavonoids

Flavonoids constitute a large and structurally diverse class of polyphenolic compounds widely distributed in plants, including fruits, seeds, flowers and other tissues. These secondary metabolites originate from phenylpropanoid pathways and encompass over 9000 identified structures. Based on differences in their chemical architecture, flavonoids are classified into several subclasses, such as flavones, flavonols, flavanones, flavanols, isoflavones, chalcones and anthocyanins. Their structural diversity is further expanded by numerous enzymatic modifications, including glycosylation, methylation, acylation, prenylation and polymerization, which significantly influence their physicochemical and biological properties [42,43,44,45].

In plants, flavonoids perform a variety of essential biological functions. They contribute to the pigmentation of flowers, fruits, and leaves and play a significant role in auxin transport, male fertility, seed development, allelopathy, and plant–microbe interactions [43,45,46,47]. Furthermore, many of them are antioxidants, scavenging ROS. and some of them may act like phytoalexins, which provide protection against various stress factors including UV light, drought, salinity, heavy metals, and cold, as well as pathogen infection and herbivory [43,44,46,47,48]. Beyond their plant-related role, they are commonly known for various health benefits in humans. They exhibit anti-oxidative, anti-inflammatory, anti-mutagenic and anti-carcinogenic properties. They can modulate cell enzyme functions and prevent cardiovascular diseases, neurodegenerative disorders, osteoporosis and cancers diseases [42,43,44,48]. Flavonoids also play a beneficial role in the prevention and management of diabetes by improving insulin sensitivity, modulating glucose metabolism and reducing oxidative stress [45,48]. These effects make them promising natural compounds for supporting metabolic health and preventing diabetes-related complications [42,45,48].

This large group of compounds is widely used in supplements, pharmaceuticals and functional food. Their widespread occurrence and diverse bioactivities have made them a major focus of research in the fields of science, biochemistry and biomedical studies [44,45,47].

The aim of this review is to critically evaluate the potential of selected flavonoids as natural inhibitors of α-glucosidase and α-amylase and to discuss their possible role as complementary agents to currently used antidiabetic therapies, including acarbose. Although the inhibitory activity of flavonoids against carbohydrate-digesting enzymes has been extensively investigated, the available evidence remains highly heterogeneous, and direct comparisons between individual compounds are often complicated by differences in enzyme origin, substrate selection, assay conditions, and approaches used for the determination of inhibitory potency. Therefore, rather than providing a descriptive summary of previously reported inhibitory activities, this review aims to integrate the available evidence with structural characteristics, structure–activity relationships, the proposed mechanisms of enzyme inhibition, and methodological limitations that may influence the reported results.

In this review, four flavonoids were selected based on their molecular structures. The aim of this manuscript is to describe the influence of an increasing number of hydroxyl groups (-OH) in the molecule on their inhibitory effects against α-glucosidase and α-amylase (i.e., chrysin—1, apigenin—2, luteolin—3, and quercetin—4).

In vitro studies were analyzed due to the broad range of available sources and the relevance of the reported data, as well as the unique context in which pharmacokinetic parameters may be less critical than usual because the site of action is located within the intestinal lumen. Unlike systemic drug targets, inhibition of α-glucosidase and α-amylase does not necessarily require high plasma concentrations of the active compound. Therefore, limited systemic bioavailability does not necessarily exclude the potential of flavonoids to exert an inhibitory effect on these intestinal enzymes. Consequently, the limited availability of pharmacokinetic data should not, in itself, preclude further clinical investigation of these compounds, as the potential clinical effect may be mediated locally within the intestinal lumen rather than through systemic exposure. In this context, systemic ADMET characteristics may be less decisive for the initial assessment of their potential efficacy than for compounds whose therapeutic targets are located in systemic tissues.

## 2. Literature Selection Methodology

This article is a narrative review. To identify relevant evidence, PubMed, Google Scholar, and ScienceDirect were searched to identify scientific publications addressing the role of flavonoids in glucose metabolism, with particular emphasis on α-amylase and α-glucosidase inhibition, the mechanisms underlying these interactions, and their potential application in the management of type 2 diabetes mellitus.

The search strategy was based on combinations of keywords related to the main topics addressed in this review. The following terms and their combinations were considered: “flavonoids” OR “flavonoid compounds” OR “polyphenols” AND “type 2 diabetes” OR “diabetes mellitus” OR “antidiabetic” AND “α-glucosidase” OR “alpha-glucosidase” OR “α-amylase” OR “alpha-amylase” AND “inhibition” OR “inhibitory activity” OR “inhibition mechanism” OR “structure–activity relationship” OR “molecular docking” OR “enzyme interaction”. Additional searches included terms related to selected compounds, including “chrysin”, “apigenin”, “luteolin”, and “quercetin”, as well as their potential effects on glucose metabolism.

The inclusion criteria were as follows: (1) peer-reviewed original research articles and review papers; (2) publications addressing flavonoids or structurally related polyphenolic compounds in the context of type 2 diabetes or glucose metabolism; (3) studies investigating the inhibition of α-glucosidase and/or α-amylase, including enzymatic, kinetic, molecular modeling, or structure–activity relationship studies; and (4) publications providing relevant information on the mechanisms of action, molecular interactions, or antidiabetic properties of flavonoids. Publications concerning selected flavonoids and their effects on glucose metabolism, insulin resistance, or related metabolic pathways were also considered when they contributed to the mechanistic background of the review.

The exclusion criteria were as follows: (1) conference abstracts, editorials, and other non-peer-reviewed publications; (2) studies unrelated to flavonoids, glucose metabolism, or the mechanisms relevant to α-glucosidase and α-amylase inhibition; (3) publications that did not provide sufficient information relevant to the objectives of this review; and (4) duplicate publications. Studies focused exclusively on unrelated biological activities of flavonoids were not considered unless they provided information directly relevant to the mechanisms discussed in this review.

The identified publications were screened by title, abstract, and keywords to assess their relevance to the scope of the review. Publications meeting the initial relevance criteria were further evaluated based on their full text. Particular attention was given to studies describing experimental conditions, inhibition mechanisms, kinetic parameters, molecular interactions, and structure–activity relationships. Relevant data and findings were subsequently extracted and incorporated into the corresponding sections of the review.

The final reference set comprised publications covering type 2 diabetes, α-glucosidase and α-amylase inhibitors, the classification and biological properties of flavonoids, as well as experimental and in silico investigations of flavonoid–enzyme interactions. The selected literature included both earlier foundational studies and recent publications, providing a comprehensive overview of the current knowledge regarding the potential role of flavonoids as modulators of carbohydrate digestion and glucose metabolism.

## 3. Flavonoids in T2DM

### 3.1. Flavonoids’ Structure–Activity Relationship in T2DM

The core structure of flavonoids is based on a 15-carbon skeleton consisting of two aromatic rings (A and B) connected by a 3-carbon bridge that typically forms a heterocyclic C ring (C6–C3–C6). Variations in the oxidation state of the central ring, as well as the number and position of substituents such as hydroxyl or methoxy groups, determine the classification of flavonoids into specific subclasses and largely account for their biological activity [43,49].

Hydroxyl groups (-OH) on aromatic rings are among the most important determinants of flavonoid biological activity. The number, position and mutual arrangement of these groups determine the ability to form hydrogen bonds with enzymatic proteins, which affects their affinity for the active sites of enzymes. For example, flavonoids with two hydroxyl groups in the 3′ and 4′ positions of the B ring, such as quercetin or luteolin, demonstrate relatively higher affinity for α-glucosidase and α-amylase and more effective inhibition of their activity in in vitro studies, compared to structures with less extensive hydroxyl substitutions [44,45,50].

In addition to the hydroxyl groups, the electronic configuration of the central ring and the conjugated bonds in the molecule influence the electron distribution in the molecule and its ability to donate electrons in redox reactions. The presence of a double bond between the carbon atoms in the central ring (C2–C3) connected to a carbonyl group (C=O) promotes electron delocalization in the π-system, which increases the compound’s antioxidant potential and its ability to neutralize ROS. Such molecules are better able to stabilize free radicals and counteract oxidative stress, which is a significant element in the pathogenesis of insulin resistance and pancreatic β-cell dysfunction in T2DM. Furthermore, this conjugation promotes a planar, stable molecular geometry, which may facilitate interactions with enzymatic proteins through π–π interactions and hydrogen bonds [45,50].

The described structure–activity relationships emphasize that not only the presence of specific functional groups but also their mutual arrangement determine the biological effectiveness of flavonoids in the context of glucose metabolism and the regulation of carbohydrate-digesting enzymes. These relationships are crucial for understanding why some flavonoids exhibit stronger PPG-lowering effects than others and how their structure can be optimized for potential therapeutic applications. The most frequently analyzed in this respect include chrysin, quercetin, apigenin and luteolin, which differ in their chemical structure and thus in their biological activity profile.

### 3.2. Flavonoids’ Mechanism of Action in T2DM

Flavonoids exert multifaceted effects in the pathophysiology of T2DM. One mechanism is the inhibition of carbohydrate-digesting enzymes, α-amylase and α-glucosidase, which slows the breakdown of starch and oligosaccharides, limiting PPG [43,44,49].

Additionally, flavonoids may increase insulin sensitivity by modulating insulin signaling pathways such as PI3K/Akt, leading to more efficient glucose uptake by peripheral tissues [45,50]. Their antioxidant properties allow for the neutralization of ROS and reduction in oxidative stress, which is one of the factors leading to pancreatic β-cell dysfunction and insulin resistance [44,47,48].

Some flavonoids also exhibit anti-inflammatory effects by reducing the production of proinflammatory cytokines such as TNF-α and IL-6, which promotes improved metabolic balance [44]. Additionally, compounds such as apigenin and chrysin influence the expression of glucose transporters (GLUT4) and enzymes involved in gluconeogenesis, regulating both glucose production in the liver and its utilization in peripheral tissues [48].

## 4. Flavonoids’ Mechanism of Action Against Digestive Enzymes

Flavonoids inhibit the activity of digestive enzymes through direct interactions with enzyme proteins, leading to disruption of their catalytic function and active site accessibility. The key mechanism is the formation of enzyme–inhibitor complexes, stabilized by hydrogen bonds, hydrophobic interactions and π–π interactions between the aromatic rings of flavonoids and the amino acid residues of the enzyme [51]. Flavonoids can act as competitive inhibitors, binding directly to the active site and competing with the substrate for access to the catalytic site. In many cases, however, a mixed or non-competitive mechanism is observed, in which these compounds also bind at allosteric sites, causing conformational changes of the enzyme and a reduction in its activity regardless of the presence of the substrate [41]. An important element of the mechanism is also the ability of flavonoids to modify the spatial structure of enzymes, which leads to a reduction in the availability of the active site or disruption of the orientation of catalytic residues. This phenomenon results from induced conformational changes and static complexation, in which one flavonoid molecule binds to one enzyme molecule, stabilizing its less active form [52]. Additionally, flavonoids may limit the digestion of carbohydrates by blocking the access of the substrate to the active site or by interacting with the substrate itself, which makes its hydrolysis by enzymes more difficult [51]. As a result, the breakdown of polysaccharides is slowed down and the rate of glucose release is reduced. The mechanism of inhibition is strongly dependent on the chemical structure of the flavonoids. The highest activity is demonstrated by aglycones containing a conjugated C2=C3 bond, a carbonyl group at the C4 position and appropriately arranged hydroxyl groups, which increase the ability to form bonds with the enzyme. At the same time, modifications such as glycosylation or methoxylation may weaken the affinity for enzymes by changing the spatial properties and polarity of the molecule [41,53].

These findings indicate that flavonoids act as multifunctional inhibitors of digestive enzymes, using both direct blocking of the active site and modulation of the enzyme structure and substrate availability, which translates into effective limitation of carbohydrate hydrolysis.

### 4.1. Enzymatic Inhibition Mechanisms of Flavonoids

Flavonoids are a broad group of naturally occurring plant-derived compounds with the ability to inhibit key digestive enzymes involved in carbohydrate metabolism, including α-amylase and α-glucosidase. These enzymes are responsible for the hydrolysis of dietary starch into absorbable monosaccharides. α-Amylase catalyzes the hydrolysis of α-1,4-glycosidic bonds in amylose and amylopectin, producing oligosaccharides such as maltose and maltotriose, which are subsequently converted into glucose by α-glucosidase. Inhibition of these enzymes reduces glucose absorption in the small intestine and attenuates PPG, thereby improving glycemic control in patients with T2DM [54,55].

Flavonoids can inhibit enzyme activity through competitive or non-competitive interactions with enzyme binding sites, thereby altering enzyme conformation and catalytic efficiency. For instance, luteolin and quercetin act as competitive inhibitors of α-amylase, whereas 3′,4′-dihydroxyflavonol exhibits non-competitive inhibition. In contrast, quercetin and 3′,4′-dihydroxyflavonol act as competitive inhibitors of α-glucosidase, while luteolin inhibits this enzyme through a non-competitive mechanism. These findings highlight the structure-dependent selectivity of flavonoid-mediated enzyme inhibition [56].

The inhibitory effects of flavonoids are reversible and primarily governed by non-covalent interactions, including hydrogen bonding and hydrophobic interactions. Kinetic analyses using the Lineweaver–Burk method have demonstrated that apigenin acts as a non-competitive inhibitor of α-amylase, whereas hispidulin, nepetin, and scutellarein inhibit this enzyme through a competitive mechanism, reducing substrate affinity [57,58]. Similarly, the mode of α-glucosidase inhibition depends on flavonoid structure. Apigenin binds non-competitively, nepetin and scutellarein act as mixed inhibitors, whereas hispidulin exhibits both inhibitory mechanisms. These differences are associated with variations in hydroxyl substitution patterns and the positions of hydroxyl groups within the flavonoid scaffold [57]. Furthermore, combinations of flavonoids and acarbose may produce synergistic inhibitory effects because these compounds act through complementary mechanisms, involving competitive and non-competitive binding at distinct enzyme sites. For example, apigenin acts as a non-competitive inhibitor, whereas acarbose binds competitively, resulting in more effective enzyme suppression than either compound alone [57,58]. The inhibitory process is also time-dependent. Extended pre-incubation of α-amylase with flavonoids has been shown to enhance inhibitory efficacy, indicating that enzyme–flavonoid binding kinetics play a decisive role in determining both the strength and duration of inhibition [59]. Collectively, these findings indicate that flavonoid-mediated inhibition of carbohydrate-hydrolyzing enzymes is a dynamic and reversible process largely governed by structural determinants.

### 4.2. Molecular Basis of Flavonoid–Enzyme Interactions

The inhibitory activity of flavonoids toward digestive enzymes depends on the spatial structure of the flavonoid scaffold, its electronic properties, and its interactions within the enzyme’s catalytic pocket. Molecular docking, a computational technique used to predict the orientation and binding affinity of small molecules toward target proteins, has provided valuable insights into these interactions. Docking studies indicate that hydroxyl groups at the C3′ and C4′ positions of the B ring, together with the carbonyl group at C4 and the C2=C3 double bond, are crucial for hydrogen bonding and π–π stacking interactions with catalytic residues of α-amylase, including Asp197, Asp300, and Glu233 [41,60].

Research indicates that quercetin and luteolin can stabilize the enzyme–inhibitor complex through the formation of hydrogen bonds and van der Waals interactions with catalytic residues, thereby preventing substrate access to the active site. However, excessive hydroxylation may disrupt π–π stacking interactions and reduce inhibitory efficiency by decreasing the overall stability of the enzyme–inhibitor complex [60]. Structural analyses have shown that hydroxylation at the C3′ and C4′ positions enhance α-amylase inhibition, whereas hydroxyl groups at C3 and C5′, as well as methoxy substitutions at C3′ and C5′, reduce inhibitory activity. These structure–activity relationships help explain why myricetin, despite forming multiple hydrogen bonds, exhibits weaker inhibitory activity than the less hydroxylated luteolin [41,60].

Moreover, molecular docking and crystallographic studies have demonstrated that flavonoid aglycones bind within the same region as acarbose, directly overlapping with key catalytic residues, whereas glycosylated flavonoids adopt an alternative binding orientation while still maintaining hydrogen bond interactions [41]. Nevertheless, molecular docking provides valuable mechanistic insights, although predicted interactions require experimental validation. A similar interaction pattern has been observed for α-glucosidase, where hydroxyl groups located on the A and B rings contribute to the formation of stable enzyme–inhibitor complexes. Binding to key active-site residues induces conformational changes in the enzyme, including disruption of α-helical structures and an increase in random coil content. These structural alterations hinder substrate access and reduce enzymatic activity [54].

At the structural level, enhanced inhibitory activity is associated with molecular planarity resulting from conjugation, hydroxylation at the C5 and C7 positions, and the presence of an o-dihydroxyl substitution pattern on the B ring. These structural features promote optimal hydrogen bond formation and hydrophobic interactions within the catalytic pocket, thereby explaining the high inhibitory potency of quercetin and luteolin [40,41,54,56,60].

Collectively, available evidence indicates that flavonoid-mediated inhibition of starch-digesting enzymes is strongly influenced by hydroxylation patterns, molecular conjugation, planarity, and binding orientation within the active site. Understanding these structure-dependent interactions provides valuable insight into the molecular basis of flavonoid activity and may support the rational design of novel antidiabetic agents [40,41,56,58,60].

To illustrate the practical implications of these structure–activity relationships, the inhibitory properties of four representative flavonoids—chrysin, apigenin, luteolin, and quercetin—are discussed below.

## 5. Comparative Analysis of Chrysin, Apigenin, Luteolin and Quercetin as Inhibitors of α-Glucosidase and α-Amylase

### 5.1. Chrysin Properties

Chrysin (5,7-dihydroxyflavone) belongs to the flavone subclass of flavonoids and is characterized by a relatively simple structure lacking substitutions in the B ring, which influences its biological activity and bioavailability. In the context of metabolic disorders, chrysin has been shown to modulate glucose metabolism by affecting the activity of digestive enzymes and regulating insulin-related signaling pathways. It has been demonstrated that chrysin can inhibit both α-amylase and α-glucosidase, leading to a reduction in PPG [38]. Additionally, it may activate AMPK signaling and improve insulin sensitivity through modulation of genes involved in glucose metabolism [61]. Its antioxidant and anti-inflammatory properties further contribute to its potential protective effects by limiting oxidative stress and pancreatic β-cell damage [62].

#### 5.1.1. Interaction with α-Glucosidase

Chrysin is among the most extensively studied flavonoids; however, literature data regarding its inhibitory potency against α-glucosidase are highly inconsistent and generally fall into two opposing groups [41].

Some studies report that chrysin exhibits stronger inhibitory activity than acarbose. One study reported an IC50 of 814.33 nM for chrysin compared with 22,800 nM for acarbose [63]. Another study reported IC50 values of 422.67 µg/mL for chrysin and 996.02 µg/mL for acarbose [64]. In contrast, other studies indicate significantly weaker activity of chrysin (IC50 = 31.33 µM vs. 3.5 µM for acarbose [65], and 375.55 µM vs. 6.38 µM [66]), or even a lack of measurable inhibitory activity against α-glucosidase [38].

These discrepancies are strongly related to differences in experimental conditions as well as structural features of the compound but also may result from differences in enzyme origin, substrate selection, assay conditions, and data processing methods. Although chrysin can bind to the enzyme, the absence of hydroxyl groups at specific positions on the B ring results in a reduced capacity to form hydrogen bonds compared with structurally more substituted flavonoids such as apigenin or luteolin [38,66]. In particular, the hydroxyl group at the C5 position is considered a key functional moiety in chrysin, enabling hydrogen bond formation and contributing to its inhibitory potential [38].

#### 5.1.2. Interaction with α-Amylase

Chrysin’s inhibitory activity against α-amylase is generally described as weak to moderate, with reported IC50 values of approximately 1.771 µM [41] or 3.89 mg/mL in kinetic studies [60]. However, one experimental study reported that chrysin (IC50 = 450.16 µg/mL) exhibited stronger inhibitory activity against α-amylase than acarbose (IC50 = 678.43 µg/mL) [64]. Enzyme kinetic analyses indicate that chrysin acts as a mixed-type inhibitor with a preference for the free enzyme, binding both to the enzyme alone and to the enzyme–substrate complex [60].

Molecular docking studies provide further insight into the interaction between chrysin and the α-amylase active site. The binding is characterized by the formation of two hydrogen bonds with the glutamine residue, as well as π interactions with tryptophan (Trp59) and tyrosine (Tyr62), which contribute to stabilization of the enzyme–inhibitor complex [60].

Similarly to α-glucosidase, the absence of hydroxyl groups at the C3′ and C4′ positions reduce inhibitory potency. Flavonoids containing these functional groups (e.g., luteolin or quercetin) can form additional hydrogen bonds with key catalytic residues such as Asp197 and Arg195, resulting in significantly stronger inhibition of α-amylase [41,60].

### 5.2. Apigenin Properties

Apigenin (4′,5,7-trihydroxyflavone) is a flavone characterized by a moderate number of hydroxyl groups, which influences its biological activity and ability to interact with enzymes. It participates in the regulation of glycemia primarily through inhibition of digestive enzymes, although its inhibitory potency is generally lower than that of quercetin [38]. An important mechanism of apigenin action involves modulation of insulin-related signaling pathways, including activation of AMPK and regulation of glucose transporter expression [67]. Moreover, apigenin exhibits anti-inflammatory effects through inhibition of NF-κB signaling, which may contribute to improved insulin sensitivity [68].

#### 5.2.1. Interaction with α-Glucosidase

Apigenin is considered a potent inhibitor of α-glucosidase [41]. Based on multiple studies, it has been reported to exhibit significantly stronger inhibitory activity against α-glucosidase than acarbose [41,69]. Reported IC50 values vary across studies, ranging from approximately 9.04 µM to lower values; however, under comparable experimental conditions, apigenin consistently demonstrates higher activity than acarbose [41,57,70].

Apigenin acts as a non-competitive inhibitor of α-glucosidase. It binds to an allosteric site without directly affecting substrate affinity, but reduces the maximum reaction velocity (Vmax). Molecular docking simulations indicate that apigenin binds within a hydrophobic pocket of the enzyme, stabilizing the complex primarily through interactions involving the hydroxyl group at the C4′ position of the B ring with residues Gly161 and Glu422. Additionally, hydrogen bonds are formed between the hydroxyl group at the C7 position of the A ring and Asp233, along with hydrophobic interactions involving residues such as Ala418 and Ile419 [56]. Other studies also report hydrogen bonding interactions with Thr306, Asn350, and Asp352 [71].

#### 5.2.2. Interaction with α-Amylase

In contrast to its activity against α-glucosidase, most studies indicate that apigenin exhibits weak to moderate inhibitory activity against α-amylase and is less potent compared with acarbose [41,58]. In one study, the IC50 value for apigenin was 21.66 µM, whereas for acarbose it was 1.66 µM [58]. However, the strong α-glucosidase inhibition combined with relatively weak α-amylase inhibition is considered therapeutically advantageous due to a reduced risk of gastrointestinal side effects such as diarrhea, flatulence, or abdominal discomfort [41].

Similarly to α-glucosidase, apigenin acts as a non-competitive inhibitor of α-amylase, interacting with a non-catalytic site where it forms hydrogen bonds with residues such as Glu493, His491, Ala489, Lys457, and Trp396. Interestingly, spectroscopic analyses have demonstrated that apigenin binding induces conformational changes in the secondary structure of α-amylase, leading to a significant reduction in catalytic activity [58].

It is also worth noting that due to its non-competitive mode of inhibition, apigenin may exhibit synergistic effects when combined with competitive inhibitors, such as other flavones (e.g., scutellarin or nepetin) and acarbose [57,58].

### 5.3. Luteolin Properties

Luteolin (3′,4′,5,7-tetrahydroxyflavone) is characterized by the presence of a catechol system in the B ring, which significantly enhances its antioxidant activity and its ability to interact with proteins. It exhibits strong inhibitory activity against both α-amylase and α-glucosidase, leading to reduced carbohydrate digestion and attenuation of PPG [38]. Luteolin also modulates signaling pathways involved in glucose metabolism, including activation of AMPK and improvement in insulin function [72]. In addition, its antioxidant and anti-inflammatory properties contribute to the protection of pancreatic β-cells and reduction in oxidative stress [73].

#### 5.3.1. Interaction with α-Glucosidase

Luteolin is widely recognized as a potent α-glucosidase inhibitor and frequently demonstrates higher inhibitory activity than acarbose [74]. Most studies report IC50 values significantly lower than those of the standard antidiabetic drug, for example 32.3 µM for luteolin compared with 815.4 µM for acarbose [74]. Other studies report IC50 values of 13.07 µM [75] and 46.00 µM [38] for luteolin, compared with 228.16 µM [75] and 606 µM [38] for acarbose.

Luteolin acts as a reversible non-competitive inhibitor and shows comparable affinity for both the free enzyme and the enzyme–substrate complex. Its binding induces conformational changes in the enzyme, including alterations in secondary structure, which lead to reduced catalytic efficiency, similar to the mechanism observed for apigenin in α-amylase inhibition [74]. Luteolin anchors within a hydrophobic pocket and forms hydrogen bonds with key amino acid residues such as Asp352, Thr306, and Asn350 [72], or alternatively interacts with Asn283 and Thr287, as well as π–π stacking interactions with Tyr286 [74].

Relative to chrysin and apigenin, luteolin exhibits stronger inhibitory activity against α-glucosidase due to the presence of a catechol moiety in the B ring, which is absent in chrysin and only partially represented in apigenin (4′-OH) [38].

#### 5.3.2. Interaction with α-Amylase

Luteolin is generally described as a weak to moderate α-amylase inhibitor and is typically considered less potent than acarbose. This dual inhibitory profile, similar to that of apigenin, is considered therapeutically beneficial, as it reduces the risk of gastrointestinal side effects associated with strong α-amylase inhibition by synthetic drugs [41].

Some studies describe luteolin as a competitive inhibitor [56], whereas others report non-competitive inhibition [76]. Luteolin binding disrupts the native conformation of the enzyme, similarly to α-glucosidase, resulting in reduced carbohydrate hydrolysis activity [58,60]. Its inhibitory activity is largely attributed to the presence of hydroxyl groups at the C3′ and C4′ positions, which enable stable hydrogen bonding with key amino acid residues in the active site [41,56]. Luteolin fits into the active site of α-amylase, forming interactions with catalytic residues such as Glu233, Asp197, and Trp59 [76], as well as Arg195 and Gln63 [56], which are directly involved in enzymatic catalysis.

### 5.4. Quercetin Properties

Quercetin (3,3′,4′,5,7-pentahydroxyflavone) is one of the most extensively studied flavonoids, characterized by potent biological activity resulting from the presence of multiple hydroxyl groups and a fully conjugated ring system. It exhibits multifaceted effects in the context of type 2 diabetes mellitus (T2DM), including inhibition of digestive enzymes as well as modulation of glucose metabolism at the cellular level. Quercetin effectively inhibits both α-glucosidase and α-amylase, thereby reducing the rate of carbohydrate digestion [38]. In addition, it enhances glucose uptake through regulation of GLUT4 expression and activation of AMPK signaling, contributing to improved insulin sensitivity [73]. Its strong antioxidant and anti-inflammatory properties further support protection against pancreatic β-cell dysfunction [77].

#### 5.4.1. Interaction with α-Glucosidase

Quercetin is considered a highly potent inhibitor of α-glucosidase and, in the majority of in vitro studies, demonstrates greater inhibitory activity than acarbose [38,41]. However, the reported inhibition kinetics are inconsistent across the literature.

Depending on experimental conditions, quercetin has been described as a competitive, non-competitive, or mixed-type inhibitor [38,41,56,78]. Several studies report very low IC50 values, including 2.6 µM [79], 8.57 µM [80], and 15 µM [38]. In contrast, other analyses report higher inhibitory concentrations, such as 163.44 µM required for 50% enzyme inhibition [78]. The weakest reported activity corresponds to 544 µg/mL for quercetin compared with 47.23 µg/mL for acarbose [81]. Despite these isolated reports, the overall evidence supports high inhibitory potency.

Quercetin binds within the catalytic pocket of α-glucosidase, blocking access of natural substrates, and its stability is maintained by an extensive network of hydrogen bonds. The 3-OH group on the C ring plays a key role by forming a hydrogen bond with the Asp349 residue and is considered a critical structural determinant of its inhibitory activity relative to luteolin and apigenin. Additionally, the catechol moiety in the B ring contributes to complex stabilization, where the 3′-OH group forms hydrogen bonds with Glu276, while the 4′-OH group interacts with Asp214 [38].

#### 5.4.2. Interaction with α-Amylase

In contrast to its activity against α-glucosidase, quercetin exhibits weak to moderate inhibitory activity against α-amylase and is generally considered less potent than acarbose, as reflected by higher IC50 values [38,78,82]. Direct comparative studies consistently demonstrate the superior inhibitory efficacy of acarbose. In one study, quercetin required a concentration of 481.26 µg/mL to achieve 50% inhibition of α-amylase, whereas acarbose achieved the same effect at 2.25 µg/mL [40]. Other studies report IC50 values of 71.49 µmol/L for quercetin versus 5.80 µmol/L for acarbose [78], as well as 270 µg/mL for quercetin compared with 32.3 µg/mL for acarbose [81]. As discussed above, this dual inhibitory profile may be considered pharmacologically advantageous.

Quercetin has been reported to act as a reversible competitive inhibitor; however, in studies involving human salivary α-amylase, its inhibition has also been described as non-competitive. Interaction of quercetin with the free enzyme may induce conformational changes in its secondary structure, resulting in reduced catalytic activity [56,81].

The active site of α-amylase forms a relatively wide V-shaped pocket in which quercetin is stabilized by π–π interactions with residues such as Tyr62 and Trp59 [56,76]. The complex is further stabilized through hydrogen bonding involving the 3′-OH and 4′-OH groups of the B ring with key residues such as Arg195 and Glu233, while the 5-OH group of the A ring interacts with Gln63 [56]. These interactions collectively limit access of natural substrates to the catalytic site. A similar binding pattern has been observed in salivary α-amylase, involving residues including Glu233, Asp197, His101, and Gln63 [76].

Furthermore, quercetin may contribute to the reduction in postprandial blood glucose levels by inhibiting intestinal glucose absorption, including modulation of the GLUT2 transporter [40,54,55].

To provide a more comprehensive interpretation of these interactions, the experimental conditions reported in the selected studies were also compared, with particular attention to factors that may influence the observed inhibitory activity.

The experimental conditions compiled in Table 1. clearly demonstrate that methodological parameters exert a critical influence on the determined inhibitory potency IC_50_ and inhibition mechanisms of chrysin, apigenin, luteolin, and quercetin against α-amylase and α-glucosidase. Among the methodological factors, the enzyme source appears to have the greatest impact on the reported inhibitory activity. Enzymes derived from Saccharomyces cerevisiae tend to be considerably more susceptible to flavonoid inhibition than those derived from mammalian or human systems, potentially resulting in several-fold differences in the reported IC_50_ values. Furthermore, parameters such as the pre-incubation time (ranging from 5 min to 2 h) and the type of substrate used (synthetic chromogens versus natural starch) significantly modulate the flavonoid–enzyme binding kinetics, directly impacting the classification of the inhibition type (competitive, non-competitive, or mixed-type).

The binding sites of chrysin, apigenin, luteolin, and quercetin with analyzed enzymes are presented in Table 2. The molecular structures of the enzymes were obtained from the RCSB Protein Data Bank (RCSB PDB) [83].

Comparison of the four flavonoids reveals a clear structure–activity relationship. Progressive hydroxylation of the flavonoid scaffold is associated with enhanced α-glucosidase inhibition. Chrysin, lacking hydroxyl substituents in the B ring, generally exhibits the weakest activity, whereas apigenin shows improved potency due to the presence of a 4′-OH group. The introduction of a catechol moiety in luteolin further enhances inhibitory activity, while the additional C3 hydroxyl group in quercetin appears to provide the most favorable interaction pattern with catalytic residues. Consequently, the overall activity trend can be summarized as Chrysin < Apigenin < Luteolin < Quercetin.

## 6. Conclusions

Flavonoids are a group of bioactive compounds with multifaceted effects that can modulate carbohydrate digestion and glucose homeostasis, particularly in the context of T2DM. The key mechanism of their activity is the inhibition of the enzymes α-amylase and α-glucosidase, which leads to slower hydrolysis of starch and oligosaccharides, thereby limiting the rate of glucose release and reducing PPG. These compounds interact both by binding to the enzyme’s active site and through allosteric interactions, which can induce conformational changes and reduce catalytic activity. This effect is significantly complemented by flavonoids’ influence on insulin-related metabolic pathways, including activation of AMPK kinase and regulation of GLUT4 transporter expression, which promotes increased glucose uptake by peripheral tissues and improved insulin sensitivity. Additionally, they exhibit antioxidant and anti-inflammatory properties, contributing to the protection of pancreatic β-cells and reducing oxidative stress and chronic inflammation, which play a significant role in the pathogenesis of metabolic disorders. The biological activity of flavonoids is closely dependent on their chemical structure, including the presence and arrangement of hydroxyl groups, ring systems, and conjugated double bonds, which determine their ability to form stable complexes with enzymes and modulate metabolic processes. In many cases, they demonstrate an inhibitory mechanism comparable to classic synthetic inhibitors such as acarbose. However, their bioavailability, pharmacokinetic properties, and actual efficacy under physiological conditions remain variable. Analysis of the available data indicates that flavonoids may constitute a valuable element of dietary and therapeutic strategies, acting as a support for pharmacological treatment and a factor in limiting glycemic disorders. Their multifaceted action, encompassing simultaneous effects on digestive enzymes, insulin signaling, and inflammatory processes, indicates a significant role for these compounds in the complex regulation of glucose metabolism. Based on the available evidence, quercetin and luteolin appear to be the most promising flavonoids for modulation of postprandial glycaemia due to their favorable structure–activity relationships and strong α-glucosidase inhibitory activity. Nonetheless it is important to acknowledge that most available evidence originates from in vitro and computational studies. Therefore, the clinical relevance of the observed inhibitory effects should be interpreted with caution until confirmed in animal and human studies.

## Figures and Tables

**Table 1:** A comparison of flavonoids properties.

Feature/Property		Chrysin	Apigenin	Luteolin	Quercetin
Chemical structure		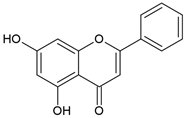	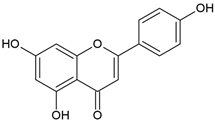	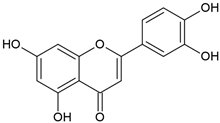	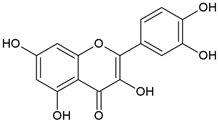
Selectivity		Low selectivity, weak inhibitor of both enzymes [41]	Selective toward α-glucosidase over α-amylase [41,58]	Strong preference for α-glucosidase over α-amylase [59,74]	Dual-target profile with preference for α-glucosidase [38,41,78,82]
α-Glucosidase Inhibition Strength	S. cerevisiae	Chrysin < Apigenin < Quercetin < Luteolin [38,57,64,74,78,81]			
	All enzyme sources *	Chrysin < Apigenin < Luteolin < Quercetin [38,41,57,63,64,65,66,69,70,74,75,78,79,80,81]			
α-Glucosidase Inhibition Mechanism		Primarily weak binding due to limited hydrogen bond formation [38,66]	Non-competitive inhibition involving hydrogen bonding in a hydrophobic pocket [57,71]	Reversible non-competitive inhibition with extensive hydrogen bonding [38,71,74]	Competitive or mixed-type inhibition with deep binding in the catalytic pocket [38,41,56,78]
α-Amylase Inhibition Strength	S. cerevisiae	Chrysin < Quercetin < Apigenin < Luteolin [58,60,64,76,81]			
	All enzyme sources **	Chrysin < Apigenin < Quercetin < Luteolin [38,40,41,58,60,64,78,81,82]			
α-Amylase Inhibition Mechanism		Mixed-type inhibition with limited hydrogen bonding and π-π interactions [41,60]	Mainly non-competitive inhibition via allosteric binding [57,58]	Competitive or non-competitive depending on conditions, with conformational effect [56,58,76]	Predominantly competitive inhibition with π-π stacking and hydrogen bonding [56,76,81]

* α-Glucosidase sources: Yeast (Saccharomyces cerevisiae); Rat Intestinal Extract (Rat Intestinal Acetone Powder); Human Intestinal Cells (Caco-2/TC7 Cell Line); Recombinant Human Intestinal Enzymes (Nt-MGAM, Ct-MGAM, Nt-SI, Ct-SI). ** α-Amylase sources: Porcine Pancreatic Amylase (PPA); Human Salivary Amylase (HSA); Human Pancreatic Amylase (HPA).

**Table 2:** Binding sites of flavonoids with α-glucosidase and α-amylase.

	α-Glucosidase	α-Amylase
Chrysin	-	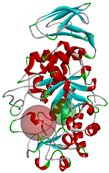
Apigenin	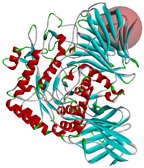	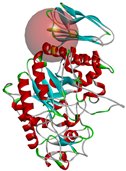
Luteolin	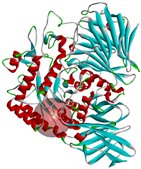	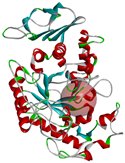
Quercetin	-	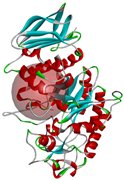

## Data Availability

No new data were created or analyzed in this study. Data sharing is not applicable to this article.

## Abbreviations

T2DM	Type 2 diabetes mellitus
PGG	Postprandial hyperglycemia
ROS	Reactive Oxygen Species
